# Enhanced virucidal activity of quaternary ammonium compound–thymol combinations: influence of mucin and mucoadhesive polymers

**DOI:** 10.1007/s00705-026-06672-8

**Published:** 2026-06-18

**Authors:** Amina Ahmady, Ian Mickleburgh, Neil Bryant, George William Carnell, Barbara A. Blacklaws

**Affiliations:** 1 Department of Veterinary Medicine, University of Cambridge, Cambridge, UK; 2 One Virology, Wolfson Centre for Global Virus Research, School of Veterinary Medicine and Science, University of Nottingham, Nottingham, UK

**Keywords:** Cetylpyridinium chloride, Benzethonium chloride, Thymol, Respiratory viruses, Influenza A, SARS-CoV-2, Virucidal activity

## Abstract

The COVID-19 pandemic highlighted the potential of oral antiseptic agents to reduce viral transmission. Strategies that enhance and sustain antiviral activity in the oral cavity may further support control of respiratory viruses. In this study, we evaluated the virucidal activity of selected antiseptic agents against influenza A virus (IAV) and severe acute respiratory syndrome coronavirus 2 (SARS-CoV-2), individually and in combination, under controlled in vitro conditions. Cetylpyridinium chloride (CPC), benzethonium chloride (BZT), thymol, and tannic acid were assessed using a quantitative suspension assay in the presence of mucin and representative mucoadhesive polymers. Dynamic light scattering and zeta potential analyses were performed to characterize interactions between active agents and mucin or polymers. CPC, BZT, and thymol exhibited dose-dependent viral inactivation. CPC reduced IAV and SARS-CoV-2 titres by approximately 4 log₁₀ at > 200 µg/mL and 25 µg/mL, respectively, while thymol achieved comparable reductions at 1000 µg/mL and 750 µg/mL. Tannic acid showed minimal virucidal activity. Notably, CPC–thymol and BZT–thymol combinations achieved > 4 log₁₀ reductions at substantially lower concentrations than individual agents, indicating enhanced virucidal activity and improved cytocompatibility. Mucin markedly attenuated the virucidal activity of CPC–thymol combinations; however, selected polymers partially preserved antiviral efficacy under mucin-rich conditions. Physicochemical analyses suggest that electrostatic, hydrophobic, and hydrogen-bonding interactions contribute to these effects. These findings demonstrate the enhanced antiviral activity of quaternary ammonium compound–thymol combinations and highlight the influence of mucosal components and macromolecules interactions in modulating oral antiseptic activity.

**Supplementary Information:**

The online version contains supplementary material available at 10.1007/s00705-026-06672-8.

## Introduction

A wide range of respiratory viruses, including severe acute respiratory syndrome coronavirus 2 (SARS-CoV-2), influenza A (IAV) and B viruses, parainfluenza virus, metapneumovirus, rhinovirus, adenovirus, and respiratory syncytial virus, replicate in the nasopharyngeal epithelium and are transmitted via respiratory droplets and aerosols [1]. The oral cavity not only serves as an entry site for these pathogens but also acts as a site of primary replication and as a reservoir that contributes to viral transmission [2].

Antiseptic mouthwashes have demonstrated in vitro virucidal activity and may reduce viral burden at mucosal surfaces, suggesting potential to reduce viral transmission and possibly alleviate disease severity [2]. Several commonly used antiseptic ingredients, such as cetylpyridinium chloride (CPC), chlorhexidine, povidone-iodine, and hydrogen peroxide, have shown varying degrees of efficacy against respiratory viruses including influenza viruses and SARS-CoV-2 [3]. Among these, CPC, a quaternary ammonium compound (QAC), exhibits particularly strong virucidal activity against enveloped viruses due to its membrane-disruptive properties [4]. Although CPC is generally considered safe at recommended concentrations (up to 0.1% in mouthwash), adverse effects (e.g., slight irritation of the gums) are concentration-dependent, supporting the need for strategies that maintain virucidal efficacy while reducing required doses [5].

One potential approach is the use of combinations of antiseptic agents to achieve enhanced virucidal effects. Synergy, defined as a virucidal effect exceeding the sum of the effects of individual agents [6], is well studied in the context of antiviral drugs, but has been less frequently investigated for antiseptic agents. Combining agents with distinct physicochemical properties or mechanisms of action may enhance virucidal performance and permit lower effective concentrations [7]. For example, CPC combined with benzydamine hydrochloride has demonstrated enhanced and more rapid inactivation of SARS-CoV-2 compared with CPC alone [8]. However, the effects of combining QACs with antiviral phytochemicals have not been systematically investigated.

Natural phytochemicals such as thymol and tannic acid possess reported antiviral activity, mediated through mechanisms including membrane disruption, protein binding, and interference with viral entry [9]. Combining QACs with such compounds may alter virucidal potency and concentration requirements, although the net effect on virucidal efficacy and cytocompatibility remains unclear.

Importantly, the virucidal performance of antiseptic agents may be significantly influenced by the complex macromolecular environment of the oral cavity. Salivary components, particularly mucin, can interact with cationic compounds and attenuate their activity. Reduced virucidal efficacy of CPC in saliva compared with buffer has been reported, potentially due to electrostatic interactions with negatively charged proteins, altered diffusion dynamics, and micelle formation [10]. Understanding how mucosal macromolecules modulate virucidal activity is therefore essential for predicting virucidal performance under physiologically relevant conditions.

In addition to endogenous salivary components, exogenous macromolecules such as mucoadhesive polymers are frequently incorporated into oral antiseptic products and may be released into saliva following application. Once present in the oral cavity, these polymers can alter the local physicochemical environment. Their defined properties, including viscosity, surface charge, and hydrophilicity, may influence diffusion dynamics and electrostatic interactions between antiseptic agents, mucosal constituents, and viral envelopes. As a result, polymers may either enhance or attenuate virucidal activity. Evaluating their effects under controlled conditions therefore provides insight into how complex macromolecular environments modulate virucidal efficacy at mucosal surfaces.

In this study, we aimed to (1) determine the virucidal activity of thymol, tannic acid, and representative QACs (CPC and benzethonium chloride) individually and in combination; (2) investigate the impact of mucin and selected mucoadhesive polymers on virucidal efficacy; and (3) characterize physicochemical interactions underlying these effects. Together, these investigations provide mechanistic insight into how combinations of antiseptic agents enhance their virucidal activity within mucin-rich macromolecular environments.

## Materials and methods

### Materials

CPC, BZT and tannic acid were dissolved in Dulbecco’s phosphate-buffered saline (DPBS) (all from Sigma-Aldrich) under aseptic conditions. Thymol (Sigma Aldrich) was dissolved in dimethyl sulfoxide (DMSO; Sigma-Aldrich). Stock solutions (10 mg/mL) of mucin (type II, from porcine stomach), sodium carboxymethyl cellulose (NaCMC; low viscosity), polyvinyl alcohol (PVA; average molecular weight 30,000–70,000 g/mol), hydroxyethyl cellulose ethoxylate quaternized (HECEQ) (all from Sigma-Aldrich) were prepared in DPBS and sterilized by autoclaving at 121 ° C for 15 min. The pH of the mucin solution was adjusted to 7.0 by the addition of the required amount of sodium hydroxide and confirmed using a digital pH meter (Eutech pH2700, Eutech Instruments Pte Ltd, Singapore). After autoclaving, the mucin solution was sonicated at room temperature for 10 min using a UW-series Ultrawave water bath sonicator (Ultrawave Ltd., Cardiff, UK), followed by centrifugation at 170 × ***g*** for 5 min using an IEC CL40R centrifuge (Thermo Fisher Scientific, Waltham, MA, USA). The supernatant was collected for subsequent experiments, and the pellet was dried in an oven and weighed to estimate mucin loss during processing. All stock solutions, whether of active or interfering compounds, were aliquoted and stored at − 25 ° C until use to maintain stability and prevent degradation.

### Cell culture

MDCK-II cells were cultured in Dulbecco’s Modified Eagle Medium (DMEM; Sigma-Aldrich), supplemented with 10% fetal bovine serum (FBS; Gibco, Thermo Fisher Scientific) and 1% penicillin/streptomycin (v/v), and incubated at 37 ° C with 5% CO₂.

For VAT cells (Vero cells expressing human ACE-2 and TMPRSS2), the culture medium, DMEM with 10% FBS and 1% penicillin/streptomycin (v/v), was additionally supplemented with 2 mg geneticin sulphate/mL (Thermo Fisher Scientific) and 200 µg hygromycin B/mL (Thermo Fisher Scientific) to maintain stable expression of ACE2 and TMPRSS2.

### Viruses

Influenza A virus (IAV; A/Puerto Rico/8/1934 [H1N1]; IAV; 8.0 × 10⁸ TCID₅₀/mL) and severe acute respiratory syndrome coronavirus 2 (SARS-CoV-2; Australia/VIC01/2020; 1.5 × 10⁸ TCID₅₀/mL) were kindly provided by Dr. Neil Bryant and Dr. George Carnell, Department of Veterinary Medicine, University of Cambridge, UK.

The medium for growth of IAV consisted of DMEM without FBS, supplemented with 0.5 µg TPCK-treated trypsin/mL (Sigma-Aldrich), and 0.2% bovine serum albumin (BSA; Sigma-Aldrich), and 1% penicillin/streptomycin (v/v). The medium for SARS-CoV-2 growth consisted of DMEM supplemented with 2% FBS and 1% penicillin/streptomycin (v/v).

### Virucidal activity

The virucidal activity of individual compounds and their combinations was assessed using a quantitative suspension test, following the method described by Steyer et al. (2021) [8]. The influence of key variables, including virus‒substance contact time (2 and 30 min) and the presence of interfering substances such as mucin and selected mucoadhesive polymers (non-ionic, anionic, and cationic), was systematically evaluated.

Test solutions of the active compound(s), with or without interfering substances, were prepared fresh in DPBS at the final concentrations indicated in Table 1 immediately prior to each experiment. Based on preliminary screening, lower concentrations of CPC and BZT were used for SARS-CoV-2 than for IAV. To initiate the assay, 0.1 mL of viral suspension (IAV or SARS-CoV-2) was mixed with 0.9 mL of the test solution and incubated at 37 °C for either 2 or 30 min.

**Table 1:** Final test concentrations (µg/mL) of compounds and combinations for IAV and SARS-CoV-2 assays

Compound/Combination	IAV	SARS-CoV-2
CPC	25–200	12.5–50
BZT	25–200	12.5–50
Tannic acid	250–1000	250–1000
Thymol	250–1000	250–1000
CPC-Thymol	25–50 CPC + 100 Thymol	5–10 CPC + 50–100 Thymol
BZT-Thymol	25–50 BZT + 100 Thymol	10–20 BZT + 50–100 Thymol

To halt the virucidal activity at the end of the contact period, an aliquot of the virus–compound mixture was immediately diluted in ice-cold maintenance medium (DMEM supplemented with either 0.2% BSA and TPCK-treated trypsin or 2% FBS). Serial ten-fold dilutions (from 10⁻¹ to 10⁻⁷) were prepared and transferred to 96-well plates containing MDCK-II or VAT cell monolayers (80 ‒ 85% confluent), depending on the virus. Plates were incubated at 37 ° C with 5% CO₂ for 48 h (IAV) or 24 h (SARS-CoV-2). Following incubation, wells were examined for cytopathic effect (CPE), and viral titres were calculated using the Spearman-Kärber method.

In addition to experiments evaluating the virucidal effect of active substances on SARS-CoV-2 and IAV, control experiments and cytotoxicity testing were also carried out.

In each experiment, virus viability was assessed by incubating the virus under identical conditions to the treatment groups, using DPBS, DPBS + mucin or DPBS + DMSO (vehicle control) for 30 min at 37 ° C. These conditions were used to confirm virus stability in the absence of active compounds and served as the reference for evaluating virucidal activity.

Each experiment included untreated cells that served as a negative control, providing information on cell viability throughout the whole incubation period. To eliminate false-positive results arising from cytotoxic effects, each experiment included control wells containing tenfold serial dilutions (10⁻¹ to 10⁻⁷) of each tested substance in the absence of virus. Cytotoxicity was evaluated morphologically at the end of the incubation period (24 or 48 h). In addition, the maximum non-cytotoxic concentration of each compound was determined by MTT assay as described under biocompatibility studies, following 24 h incubation for VAT cells and 48 h for MDCK-II cells.

All experimental conditions were analysed in duplicate within each assay run (technical replicates), and each experiment was repeated independently three times (biological replicates).

### Biocompatibility studies

The MTT assay was employed for two complementary purposes:

(1) to determine the maximum non-cytotoxic concentration of each antiseptic compound to exclude cytotoxic interference in virucidal assays, and (2) to evaluate and compare the short-term biocompatibility of individual and combined antiseptic agents at therapeutically relevant concentrations.

To determine maximum non-cytotoxic concentration, MDCK-II and VAT cells were exposed to CPC (0.125–20 µg/mL), BZT (0.125–20 µg/mL), thymol (2.5–100 µg/mL), and tannic acid (2.5–100 µg/mL) for 48 h and 24 h, respectively. The incubation times corresponded to those used in virucidal assays for each cell line, ensuring comparable exposure conditions.

For biocompatibility assessment, cells were exposed for 30 min to CPC (3.1–200 µg/mL), BZT (3.1–200 µg/mL), and thymol (50–500 µg/mL), alone or in combination (25–50 µg CPC or BZT/mL with 100 µg thymol/mL).

MDCK-II and VAT cells were seeded in 96-well plates at densities of 3.0 × 10⁴ and 1.5 × 10⁴ cells/well, respectively, and incubated overnight at 37 °C in a 5% CO₂ atmosphere to form confluent monolayers. After treatment for the specified duration, cells were washed with DPBS and incubated with MTT solution (0.5 mg/mL in culture medium) for 3 h at 37 °C in the dark. Formazan crystals were solubilized with 0.1 mL DMSO, and absorbance was measured at 630 nm using a microplate reader (800 TS, BioTek Instruments, USA).

Experiments were conducted in duplicate wells and repeated independently three times. Cell viability was expressed as a percentage relative to untreated control cells.

### Physicochemical characterisation of antiseptic–mucin–polymer systems

#### pH measurement

The pH of the blank and drug-loaded solutions (50 µg CPC/mL and 100 µg thymol/mL) with or without mucin and/or polymers was measured using a digital pH meter (Eutech pH2700, Eutech Instruments Pte Ltd, Singapore). For each solution, three measurements (*n* = 3) were recorded.

#### Dynamic light scattering and zeta potential analysis

Dynamic light scattering (DLS) and zeta potential measurements were performed to investigate potential interactions between mucin, antiseptic agents, and polymers. A NanoBrook Omni instrument (Brookhaven Instruments, Holtsville, NY, USA) was used to determine the hydrodynamic diameter and zeta potential of the particles. Samples were prepared following the same protocol as in the virucidal studies and incubated at 37 °C in a water bath for 30 min. Subsequently, they were diluted 1:20 in DPBS prior to measurement. DLS measurements were carried out at 25 °C. Zeta potential was determined using the Phase Analysis Light Scattering (PALS) technique, and values were calculated by the instrument software based on the Smoluchowski approximation. The dilution step was necessary to reduce viscosity and enable reliable measurement, particularly for polymer-containing systems. While dilution and measurement at 25 °C may influence mucin–compound–polymer interactions compared to incubation conditions (37 °C), all samples were analysed under identical conditions to allow comparative assessment. Each sample was analysed in triplicate, and results are reported as mean ± standard deviation.

### Statistical analysis

Statistical analyses were performed using GraphPad Prism version 10.6.0. Data are presented as mean ± standard deviation (SD). Comparisons between two groups were performed using Student’s t-test. For multiple group comparisons, one-way ANOVA was used followed by Dunnett’s test for comparisons between each treatment group and the control group, or Tukey’s multiple comparisons test for all pairwise comparisons among groups, as appropriate. These post hoc tests were used to account for multiple comparisons and control the family-wise error rate. Differences were considered statistically significant at *p* < 0.05.

For datasets containing values at the limit of detection, these values were assigned the corresponding detection-limit value and included in statistical analyses where applicable.

## Results

### Virucidal activity

#### Control procedures

**Fig. 1:**
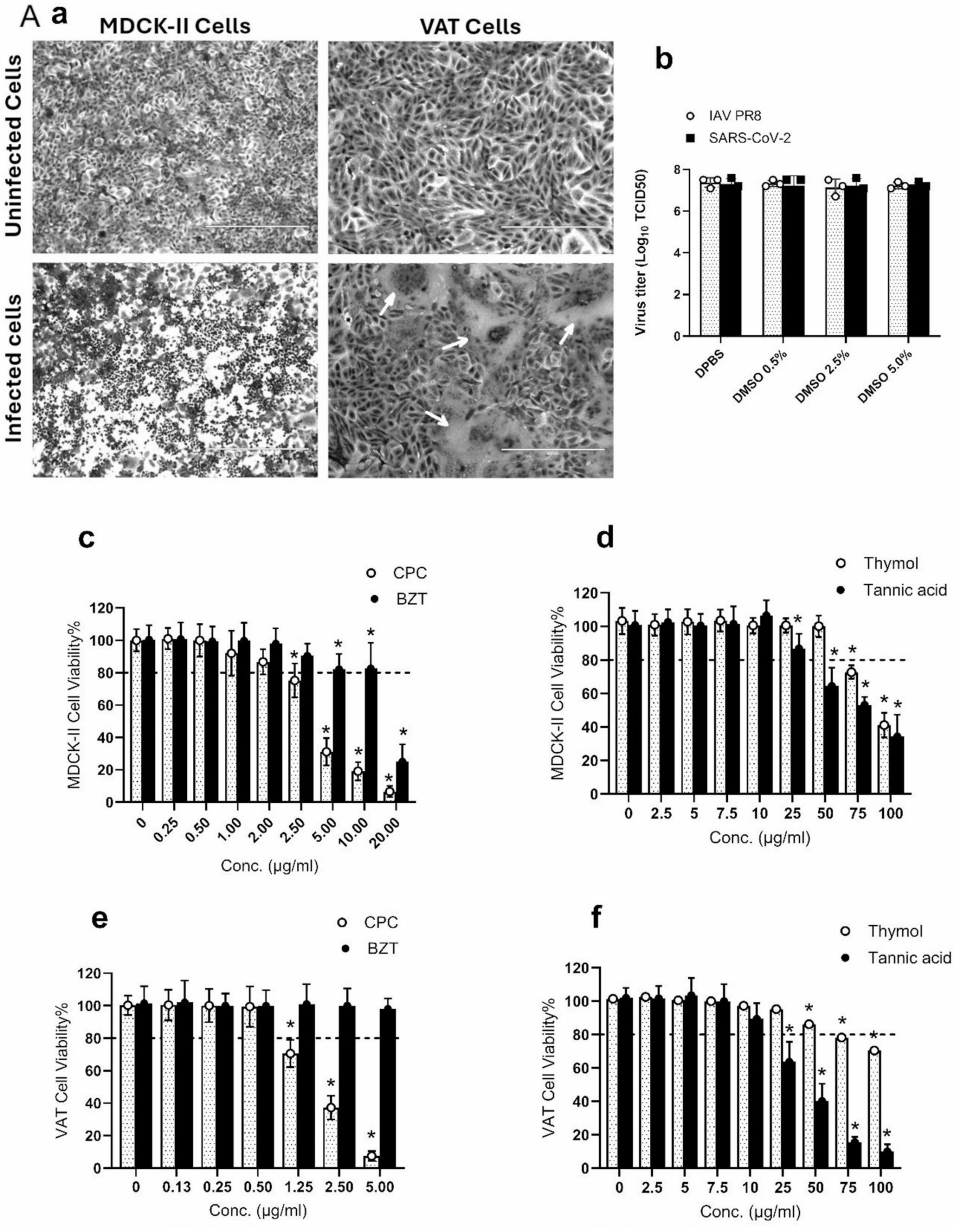
(**a**) Brightfield images of uninfected and virus-infected MDCK-II and VAT cells captured using the EVOS FL Cell Imaging System (Thermo Fisher Scientific, Waltham, MA, USA). (**b**) Comparative viral titres of IAV and SARS-CoV-2 following 30 min incubation with DPBS alone or DPBS containing different concentrations of DMSO. (**c**–**d**) Cell viability (%) of MDCK-II cells after 48 h exposure to varying concentrations of CPC or BZT (**c**) and thymol or tannic acid (**d**). (**e**–**f**) Cell viability (%) of VAT cells after 24 h exposure to varying concentrations of CPC or BZT (**e**) and thymol or tannic acid (**f**). Data represent mean ± SD (*n* = 6). Statistical analysis was performed using one-way ANOVA followed by Dunnett’s multiple comparisons test, comparing each treatment group with the corresponding control. Statistical significance is indicated in the graphs (* *p* < 0.05)

Both viruses produced characteristic cytopathic effects (CPEs) in their respective host cells, confirming assay reliability. IAV-infected MDCK-II cells exhibited cell rounding and partial monolayer detachment, whereas SARS-CoV-2 infected VAT cells exhibited prominent syncytia, a typical coronavirus-induced CPE in VAT cells expressing both ACE2 and TMPRSS2 (Fig. 1a).

The presence of DMSO at concentrations between 0.5% and 5% did not significantly affect IAV or SARS-CoV-2 infectivity (*p* > 0.05), confirming its suitability as a solvent control (Fig. 1b).

Cytotoxic effects were assessed microscopically by examining treated, virus-free control wells for cell deformation and monolayer disruption. In addition, cell viability was quantified using the MTT assay to confirm the maximum non-cytotoxic concentration (Fig. 1c–f). Cytotoxicity was observed for each active substance, starting from 2.5 to 1.25 µg CPC/mL in MDCK-II or VAT cells respectively, 5 µg BZT/mL in MDCK-II cells only (no cytotoxicity was seen in VAT cells), 75 or 50 µg thymol/mL in MDCK-II or VAT cells respectively, and 25 µg tannic acid/mL in MDCK-II or VAT cells respectively. Therefore, to ensure accurate assessment of viral infectivity, only dilutions of the virus–compound mixtures ranging from 10⁻² to 10⁻⁷ were included in the final analyses which were below the cytotoxic concentrations of the compounds.

#### Virucidal effect of individual active compounds in different concentrations

**Fig. 2:**
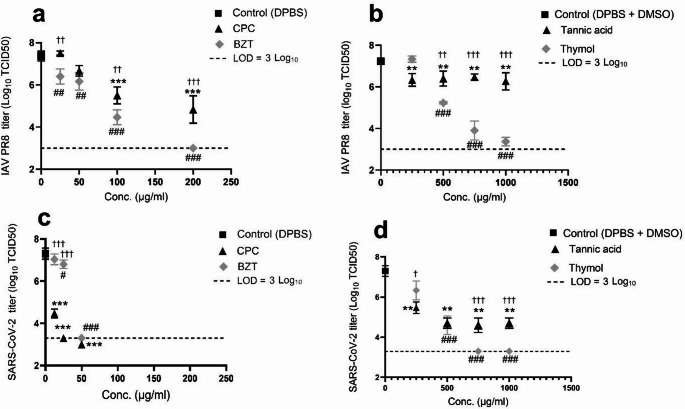
Viral titres of IAV and SARS-CoV-2 following a 30 min exposure to different concentrations of antiseptic agents (**a**–**b**) IAV titres after treatment with (**a**) CPC and BZT, and (**b**) thymol and tannic acid; **(c–d)** SARS-CoV-2 titres after treatment with (**c**) CPC and BZT, and (**d**) thymol and tannic acid. Data points at the limit of detection (LOD; 3.0 or 3.3 log₁₀TCID₅₀/mL) represent samples below assay sensitivity; actual titres may be lower. For statistical analysis, values below the LOD were conservatively assigned the LOD value. Data represent mean ± SD from six replicates (*n* = 6). Statistical analysis was performed using unpaired Student’s t-test to compare CPC and BZT at equivalent concentrations for each virus. One-way ANOVA followed by Dunnett’s multiple comparisons test was used to compare each concentration with the corresponding untreated control within each treatment group. *P < 0.05, **P < 0.01, ***P < 0.001 vs. untreated control (CPC/tannic acid); #*P* < 0.05, ##*P* < 0.01, ###*P* < 0.001 vs. untreated control (BZT/thymol); †*P* < 0.05, ††*P* < 0.01, †††*P* < 0.001 for comparisons between CPC vs. BZT or tannic acid vs. thymol at equivalent concentrations

The direct virucidal efficacy of CPC, BZT, thymol, and tannic acid against IAV and SARS-CoV-2 was evaluated following 30 min of exposure at varying concentrations (Fig. 2). Overall, a concentration-dependent reduction in viral titre was observed for CPC, BZT, and thymol, whereas tannic acid exhibited only limited activity. Notably, SARS-CoV-2 was generally more sensitive to the tested compounds than IAV.

CPC and BZT substantially reduced infectivity of both viruses (*p* < 0.05) (Fig. 2a and c). For instance, BZT reduced IAV and SARS-CoV-2 titres by ~ 4 log₁₀ TCID₅₀/mL at 200 µg/mL and 50 µg/mL, respectively, indicating effective virucidal activity. BZT showed stronger activity than CPC against IAV at equivalent concentrations (*p* < 0.05), producing 2.9 ± 0.2 log₁₀ TCID₅₀/mL reduction at 100 µg/mL, whereas CPC achieved 1.9 ± 0.2 log₁₀ TCID₅₀/mL reduction at the same concentration. Against SARS-CoV-2, CPC showed significantly greater activity than BZT at 12.5 µg/mL (*p* < 0.05), reducing titres by approximately 2.8 ± 0.1 log₁₀ TCID₅₀/mL, whereas BZT showed minimal activity at this concentration.

The effects of thymol and tannic acid are shown in Fig. 2b and d. Thymol displayed potent virucidal activity, lowering IAV and SARS-CoV-2 titres by ~ 4 log₁₀ TCID₅₀/mL at 1000 µg/mL and 750 µg/mL, respectively (*p* < 0.05). In contrast, tannic acid produced only modest virucidal activity against IAV, reducing titres by ~ 1.0 log₁₀ TCID₅₀/mL reduction across 250–1000 µg/mL (*p* < 0.05 vs. control), with no clear concentration-dependent effect. For SARS-CoV-2, reductions were larger (~ 1.8 log₁₀ TCID₅₀/mL at 250 µg/mL increasing to ~ 2.5–2.7 log₁₀ TCID₅₀/mL by 500 µg/mL) (*p* < 0.05 vs. control), but activity plateaued thereafter, suggesting a maximal effect was reached within the tested range.

Together, these results demonstrate strong and dose-dependent virucidal activity of CPC, BZT, and thymol. In contrast, tannic acid exhibited only modest to moderate effects under tested conditions, with no consistent concentration-response relationship.

#### Virucidal effect of active compounds in combination

Since tannic acid did not exhibit appreciable virucidal activity, it was excluded from further experiments. The virucidal efficacy of CPC–thymol and BZT–thymol combinations against IAV and SARS-CoV-2 was next assessed after 30 min of exposure to evaluate any potentiated activity (Tables 2 and 3).

**Table 2:** Reduction in IAV viral titre (∆log₁₀ TCID₅₀/mL) following treatment with CPC, BZT, thymol, and their combinations. Data are shown for 30- and 2-minute exposures in DPBS, and for 30-minute exposures in DPBS supplemented with mucin (2 mg/mL). For statistical analysis, values below the LOD were conservatively assigned the LOD value. Data represent mean ± SD (*n* = 6). Statistical analysis was performed using unpaired Student’s t-test for pairwise comparisons between matched experimental conditions (including DPBS vs. mucin-supplemented medium and different exposure times). One-way ANOVA followed by Dunnett’s multiple comparisons test was used to compare each treatment with the corresponding untreated control within each experimental condition

	IAV titre reduction (∆log_10_ TCID_50_/mL)		
	DPBS		DPBS + Mucin (2 mg/mL)
	30 min exposure	2 min exposure	30 min exposure
CPC (200 µg/mL)	2.5 ± 0.9*	0.4 ± 0.5^#^	1.8 ± 0.4*^#^
BZT (200 µg/mL)	> 4.4^a^*	1.3 ± 0.6*^#^	2.8 ± 0.3*^#^
Thymol (750 µg/mL)	3.5 ± 0.7*	1.6 ± 0.2*^#^	2.8 ± 0.2*
CPC (50 µg/mL) + thymol (100 µg/mL)	> 4.4*	4.1 ± 0.2*	3.9 ± 0.2*
CPC (25 µg/mL) + thymol (100 µg/mL)	> 4.4*	2.6 ± 0.3*^#^	1.1 ± 0.5*^#^
BZT (50 µg/mL) + thymol (100 µg/mL)	> 4.4*	> 4.4*	3.1 ± 0.4*^#^
BZT (25 µg/mL) + thymol (100 µg/mL)	4.1 ± 0.5*	2.2 ± 0.7*^#^	1.7 ± 0.3*^#^

^a^ >4.4 indicates a reduction in titre greater than the limit of detection.

* Statistically significant reduction in viral titre compared with the corresponding untreated control (*p* < 0.05).

^#^ Significantly different from the corresponding treatment under DPBS conditions with 30 min exposure (*p* < 0.05)

**Table 3:** Reduction in SARS-CoV-2 viral titre (∆log₁₀ TCID₅₀/mL) following treatment with CPC, BZT, thymol, and their combinations. Data are shown for 30- and 2-minute exposures in DPBS, and for 30-minute exposures in DPBS supplemented with mucin (2 mg/mL). For statistical analysis, values below the LOD were conservatively assigned the LOD value. Data represent mean ± SD (*n* = 6). Statistical analysis was performed using unpaired Student’s t-test for pairwise comparisons between matched experimental conditions (including DPBS vs. mucin-supplemented medium and different exposure times). One-way ANOVA followed by Dunnett’s multiple comparisons test was used to compare each treatment with the corresponding untreated control within each experimental condition

	SARS-CoV-2 titre reduction (∆log_10_ TCID_50_/mL)		
	DPBS		DPBS + Mucin (2 mg/mL)
	30-min exposure	2-min exposure	30-min exposure
CPC (25 µg/mL)	> 4^a^*	0.9 ± 0.3^#^	1.5 ± 0.2*^#^
BZT (50 µg/mL)	> 4*	0.4 ± 0.3^#^	2.1 ± 0.2*^#^
Thymol (500 µg/mL)	2.7 ± 0.5*	1.1 ± 0.5*^#^	2.7 ± 0.2*
CPC (10 µg/mL) + thymol (50 µg/mL)	> 4*	> 4*	1.4 ± 0.3*^#^
CPC (5 µg/mL) + thymol (100 µg/mL)	> 4*	-^b^	0.9 ± 0.4^#^
BZT (20 µg/mL) + thymol (50 µg/mL)	> 4*	2.4 ± 1.0*^#^	1.6 ± 0.2*^#^
BZT (10 µg/mL) + thymol (100 µg/mL)	> 4*	-	1.2 ± 0.1*^#^

^a^ >4 indicates a reduction in titre that is greater than the detection limit.

^b^ – indicates that the combination was not tested under the 2 min exposure conditions.

* Statistically significant reduction in viral titre compared with the corresponding untreated control (*p* < 0.05).

^#^ Significantly different from the corresponding treatment under DPBS conditions with 30 min exposure (*p* < 0.05)

All tested combinations produced notable reductions in viral titre, achieving > 4 log₁₀ TCID₅₀/mL reductions (*p* < 0.05 vs. control). For IAV, the CPC–thymol pairs (25–50 µg CPC/mL with 100 µg thymol/mL) reduced viral titres by > 4.4 log₁₀, whereas the individual compounds at these concentrations had little to no effect (e.g. 50 µg CPC/mL: <1 log₁₀ TCID₅₀/mL reduction; 250 µg thymol/mL: no effect). Similarly, all BZT–thymol combinations surpassed the activity of either compound alone, consistently reducing titres by > 4 log₁₀ TCID₅₀/mL.

A comparable pattern was observed for SARS-CoV-2, although lower compound concentrations were sufficient to show good antiviral activity. CPC (5–10 µg/mL) or BZT (10–20 µg/mL) paired with thymol (50–100 µg/mL) resulted in > 4 log₁₀ TCID₅₀/mL titre reductions, far exceeding the effect of each compound when tested individually.

Together, these results demonstrate that CPC–thymol and BZT–thymol combinations markedly enhance virucidal activity, permitting effective viral inactivation at lower doses than single agents.

#### Virucidal effect of active compounds in short exposure times

To investigate the impact of exposure time on virucidal activity, virus suspensions were treated with individual compounds or their combinations for either 2 or 30 min. The results are summarized in Table 2 (IAV) and 3 (SARS-CoV-2) as reductions in viral titre (∆log₁₀ TCID₅₀/mL), representing the extent of virus inactivation.

Overall, for both virus species, all compounds were more effective after 30 min than 2 min of exposure. However, the magnitude of time dependency varied among the tested compounds and their combinations.

For IAV, significant differences in virucidal activity were observed for individual compounds at equivalent concentrations between 30 and 2 min of exposure (*p* < 0.05). Each compound was strongly active at 30 min but showed only limited effects at 2 min (≤ 1.6 log₁₀ TCID₅₀/mL reduction). In contrast, the combinations retained high potency even at short contact times: CPC or BZT (50 µg/mL) with thymol (100 µg/mL) reduced titres by > 4 log₁₀ TCID₅₀/mL after 2 min, and CPC or BZT (25 µg/mL) with thymol (100 µg/mL) achieved 2.6 and 2.2 log₁₀ TCID₅₀/mL reduction at 2 min.

A similar pattern was observed for SARS-CoV-2. While CPC (25 µg/mL), BZT (50 µg/mL), and thymol (500 µg/mL) each produced strong reductions after 30 min, their activity at 2 min was minimal (*p* < 0.05) (≤ 1 log₁₀ TCID₅₀/mL). By contrast, CPC (10 µg/mL) with thymol (50 µg/mL) reduced titres by > 4 log₁₀ TCID₅₀/mL within 2 min, and BZT (20 µg/mL) with thymol (50 µg/mL) produced 2.4 ± 1.0 log₁₀ TCID₅₀/mL reduction at 2 min.

Together, these findings show that while CPC, BZT, and thymol alone required extended contact times to achieve ≥ 4 log₁₀ reductions in viral titre, combining CPC or BZT with thymol markedly improved virucidal activity against both IAV and SARS-CoV-2, supporting their potential for inclusion in fast-acting antiviral formulations.

#### Virucidal effect of active compounds in the presence of interfering substances: mucin

To assess the impact of interfering substances commonly found in biological fluids, the virucidal activity of CPC, BZT, thymol, and their combinations was evaluated in the presence of mucin (2 mg/mL), a major glycoprotein component of saliva (Tables 2 and 3).

The presence of mucin affected the virucidal activity of individual tested compounds in different ways. In the case of CPC and BZT (200 µg/mL) the virucidal activity markedly decreased in the presence of mucin (*p* < 0.05). For IAV, the ∆log₁₀ TCID_50_/mL of CPC and BZT (200 µg/mL) declined from 2.5 ± 0.9 to 1.8 ± 0.4 and from > 4.4 to 2.8 ± 0.3, respectively. A similar pattern was observed for SARS-CoV-2, where CPC (25 µg/mL) and BZT (50 µg/mL) both showed a > 2 log₁₀ TCID_50_/mL increase in virus titre in the presence of mucin compared to DPBS. In contrast, thymol appeared less sensitive to interference by mucin, with only modest to no reductions in effect (*p* > 0.05) (e.g. from 3.5 ± 0.7 to 2.8 ± 0.2 log₁₀ TCID_50_/mL for IAV, and from 2.7 ± 0.5 to 2.7 ± 0.2 log₁₀ TCID_50_/mL for SARS-CoV-2), suggesting greater resistance to inactivation.

CPC–thymol and BZT–thymol combinations were also substantially impaired by mucin. For IAV, multiple combinations were tested at different concentrations (25–50 µg CPC or BZT/mL with 100 µg thymol/mL). The extent of loss of activity changed with concentration of CPC or BZT. Combinations containing higher concentrations of CPC or BZT (50 µg/mL) retained appreciable activity (e.g. 50 µg CPC/mL + 100 µg thymol/mL: 4.1 ± 0.1 log₁₀ TCID_50_/mL reduction in IAV titer) with no statistically significant difference compared with the corresponding non-mucin condition (*p* > 0.05). In contrast, lower concentration combinations exhibited stronger interference by mucin (e.g. 25 µg CPC/mL + 100 µg thymol/mL: 1.1 ± 0.5 log₁₀ TCID_50_/mL reduction in IAV titer), which was significantly reduced compared with the equivalent condition without mucin (*p* < 0.05). A similar trend was seen for BZT–thymol combinations. These findings suggest that mucin binds or otherwise neutralizes a portion of CPC and BZT, reducing their effective free concentrations; at higher doses, enough active compound remains available to inactivate the virus.

Overall, mucin strongly interferes with the virucidal activity of CPC, BZT, and their combinations with thymol, while thymol shows greater stability in the presence of this salivary component. These results highlight the importance of considering physiological conditions when evaluating potential antiviral formulations.

#### Virucidal effect of active compounds in the presence of interfering substances: mucoadhesive polymers

This part of the study focused on CPC, which is included on the U.S. FDA’s Generally Recognized As Safe (GRAS) list and approved by regulatory authorities as an oral antiseptic. To assess the influence of common polymers used in mucoadhesive formulations on its virucidal activity, CPC–thymol combinations were tested in the presence of representative polymers; PVA, NaCMC, and HECEQ, at 5 mg/mL, both with and without mucin (2 mg/mL).

**Fig. 3:**
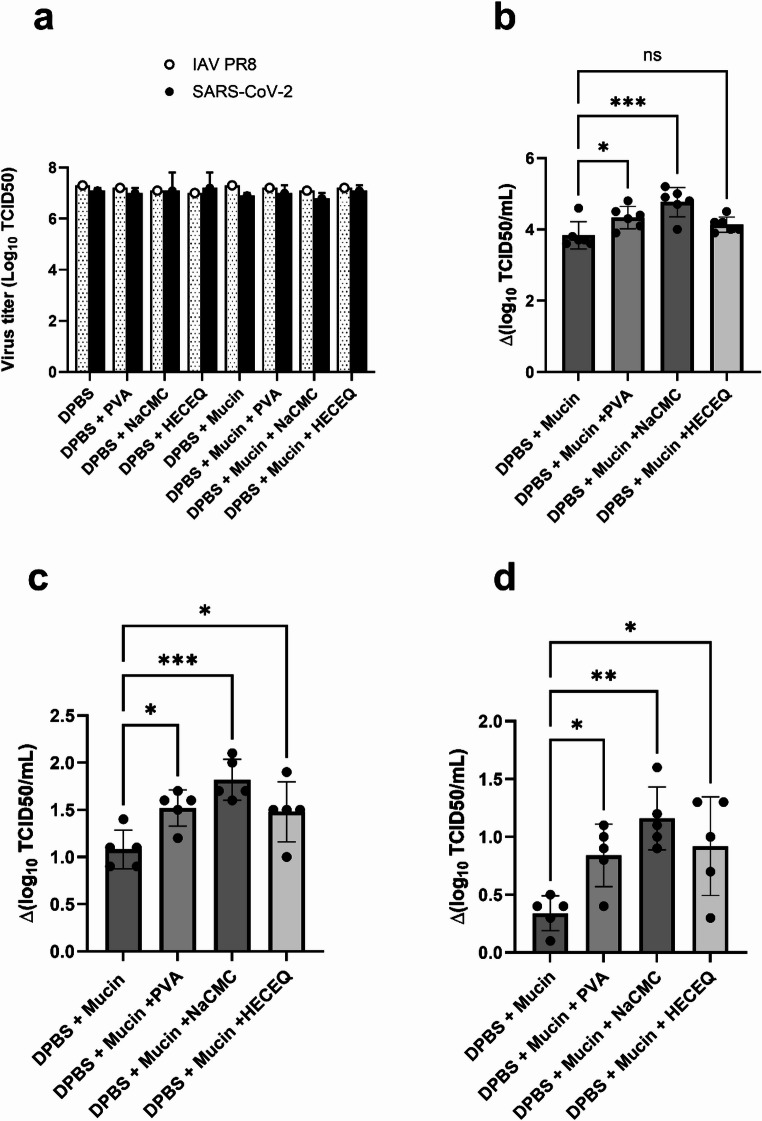
Effect of mucin and mucoadhesive polymers on CPC–thymol virucidal activity. (**a**) Viral titres of IAV and SARS-CoV-2 in DPBS with or without mucin and/or polymers. (**b**–**d**) Reduction in viral titre (∆log₁₀ TCID₅₀/mL from controls) after exposure to CPC + thymol combinations in mucin or mucin–polymer suspensions: (**b**) IAV, 50 µg CPC/mL + 100 µg thymol/mL; (**c**) IAV, 25 µg CPC/mL + 100 µg thymol/mL; (**d**) SARS-CoV-2, 10 µg CPC/mL + 50 µg thymol/mL. All incubations were for 30 min. Data represent mean ± SD (*n* = 5). Statistical analysis was performed using one-way ANOVA followed by Dunnett’s test for comparisons against the corresponding control group. Statistical significance is indicated in the graphs as *p < 0.05, **p < 0.01, and ***p < 0.001

Control experiments confirmed that viral titres of both IAV and SARS-CoV-2 remained unchanged after incubation in DPBS, DPBS supplemented with polymers (PVA, NaCMC, HECEQ), mucin, or mucin–polymer mixtures, demonstrating that these excipients have no intrinsic antiviral activity under the tested conditions (Fig. 3a). Furthermore, when CPC–thymol combinations were tested in DPBS with polymers, virucidal activity was comparable to that observed in DPBS alone (a decrease of > 4.4 log_10_ TCID_50_/mL for IAV and > 4 log_10_ TCID_50_/mL for SARS-CoV-2), indicating that the polymers themselves did not sequester or inactivate the antiseptic compounds (Table S1).

In DPBS supplemented with mucin alone, CPC–thymol treatment resulted in mean reductions of 3.8 ± 0.4 log₁₀ TCID₅₀/mL and 1.1 ± 0.2 log₁₀ TCID₅₀/mL for IAV at higher and lower CPC concentrations, respectively, and 0.4 ± 0.2 log₁₀ TCID₅₀/mL for SARS-CoV-2. The inclusion of polymers generally enhanced virucidal activity compared with mucin alone (*p* < 0.05) (Fig. 3b‒d). In particular, NaCMC-containing treatments produced the greatest increases in viral titre reduction across all conditions, reaching 4.8 ± 0.2 TCID₅₀/mL for IAV (50 µg CPC/mL), 1.8 ± 0.2 TCID₅₀/mL for IAV (25 µg CPC/mL), and 1.2 ± 0.3 TCID₅₀/mL for SARS-CoV-2. PVA and HECEQ also improved virucidal efficacy relative to mucin alone, though to a lesser extent. Overall, these results indicate that polymer inclusion can partially mitigate mucin-associated attenuation of CPC–thymol virucidal activity.

### Biocompatibility assessment and safety profile of antiseptic compounds

**Fig. 4:**
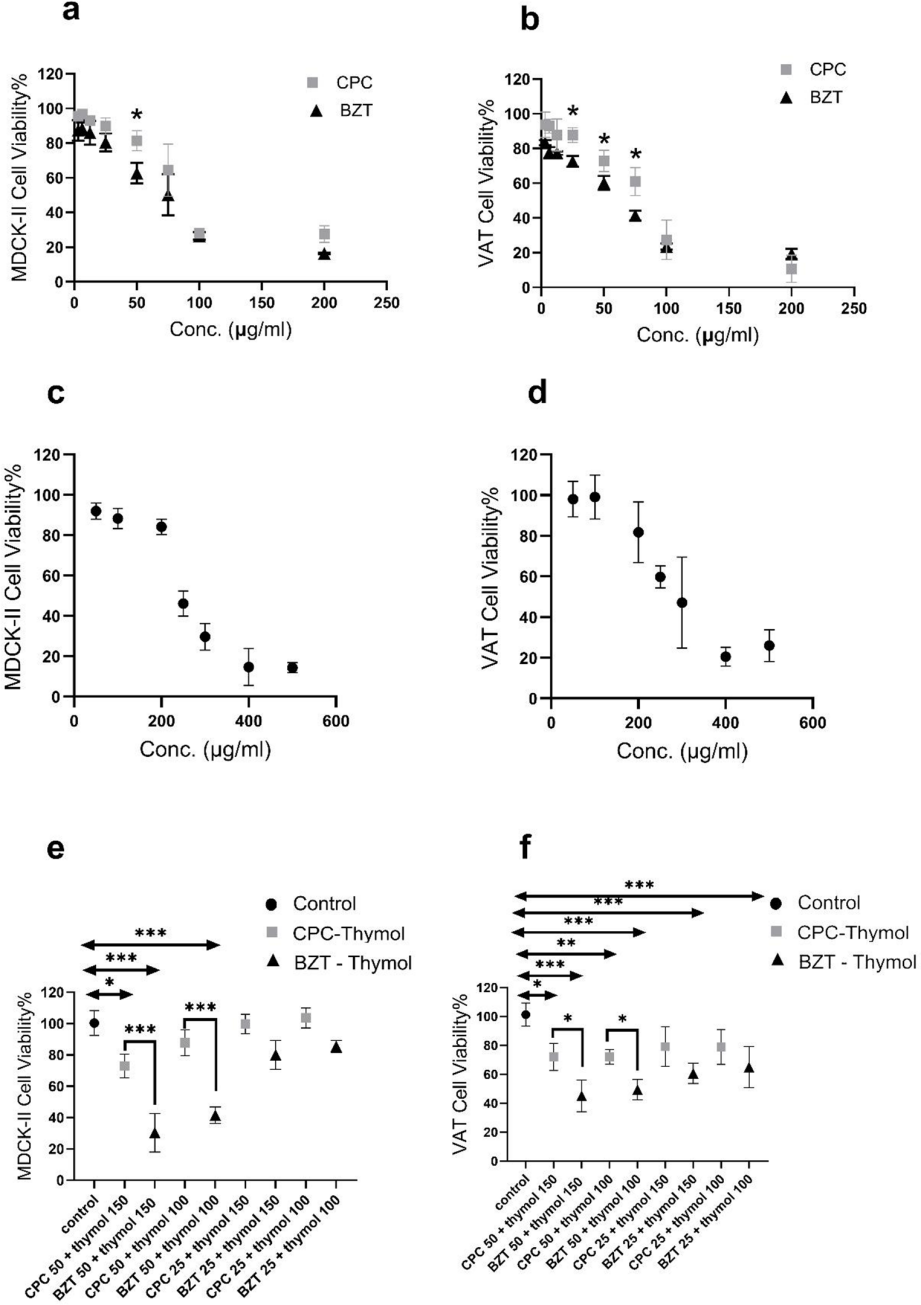
Cytotoxicity of CPC, BZT, thymol, and their combinations in MDCK-II and VAT cells after 30-minute exposure. Dose-dependent effects of CPC and BZT on (**a**) MDCK-II cells and (**b**) VAT cells; thymol on (**c**) MDCK-II cells and (**d**) VAT cells; and CPC–thymol and BZT–thymol combinations on (**e**) MDCK-II cells and (**f**) VAT cells. Data represent mean ± SD (*n* = 3). Statistical analysis was performed using Student’s t-test to compare CPC versus BZT and CPC–thymol versus BZT–thymol at selected equivalent concentrations. Statistical significance is indicated in the graphs as * *p* < 0.05, ** *p* < 0.01, and *** *p* < 0.001

To evaluate the cytotoxicity of the tested compounds, cell viability was assessed in MDCK-II and VAT cells following exposure to increasing concentrations of CPC, BZT, thymol, and their combinations (Fig. 4).

As shown in Fig. 4a and b, both CPC and BZT induced a clear, dose-dependent reduction in cell viability. At 100 µg/mL, cell survival declined to approximately 30% for CPC and 25% for BZT, while at 200 µg/mL both compounds reduced viability to below 20%, indicating pronounced cytotoxicity at virucidal concentrations. Direct comparison revealed that CPC generally exhibited a slightly more favourable safety profile, maintaining higher viability than BZT at equivalent concentrations, however, this difference was not statistically significant at most tested concentrations. In contrast, thymol displayed a relatively favourable cytotoxicity profile, sustaining > 80% viability up to ~ 200 µg/mL, although viability declined sharply to < 20% at concentrations ≥ 400 µg/mL (Fig. 4c and d). Overall, MDCK-II and VAT cells exhibited broadly comparable sensitivity to the individual compounds across the tested concentration ranges (*p* > 0.05).

Regarding the CPC–thymol and BZT-thymol combinations (Fig. 4e and f), CPC-thymol exhibited a less toxic profile, maintaining > 80% viability across most tested conditions. The exception was the highest-dose combination (50 µg CPC/mL + 150 µg thymol/mL), for which MDCK-II viability decreased significantly to ~ 72% compared with untreated control (*p* < 0.05). In contrast, BZT–thymol combinations generally showed lower viability than CPC–thymol combinations. This difference was statistically significant at the highest tested concentration (i.e., 50 µg QAC/mL), indicating a comparatively less favourable biocompatibility profile (*p* < 0.05). Importantly, VAT cells consistently showed 10–15% lower viability than MDCK-II cells across the tested combinations, although the relative patterns were similar in both models (e.g. CPC–thymol generally less toxic than BZT–thymol and higher viability with lower BZT concentrations).

### Physicochemical characterisation of antiseptic–mucin–polymer systems

#### pH Measurement

The pH of all tested systems remained within the physiological range (6.8–7.4) (Table 4). Addition of the CPC–thymol combination at the highest tested concentration did not result in any meaningful change in pH compared with the corresponding blank controls (*p* > 0.05). Slight reductions in pH were observed following the inclusion of mucin and polymers; however, only PVA caused a statistically significant decrease in medium pH (*p* < 0.05). Overall, these changes were minimal and remained within physiologically acceptable limits.

**Table 4:** pH values of the tested systems. Data represent mean ± SD (*n* = 3). Statistical analysis was performed using one-way ANOVA followed by Dunnett’s multiple comparisons test to assess the effect of mucin and polymer additives on pH within each system (DPBS alone or DPBS loaded with CPC (50 µg/mL) + thymol (100 µg/mL))

Medium	Blank	CPC (50 µg/mL) + Thymol (100 µg/mL)
DPBS	7.35 ± 0.2	7.38 ± 0.3
DPBS + Mucin^a^	7.20 ± 0.1	7.24 ± 0.2
DPBS + PVA^b^ + Mucin	6.80 ± 0.2*	6.82 ± 0.2*
DPBS + HECEQ^c^ + Mucin	6.98 ± 0.1	7.05 ± 0.1
DPBS + NaCMC^d^ + Mucin	7.10 ± 0.2	7.20 ± 0.2

^a^ Mucin concentration: 2 mg/mL

^b^ PVA: polyvinyl alcohol;

^c^ HECEQ: Hydroxy ethyl cellulose ethoxylate quaternized;

^d^ NaCMC: Sodium carboxymethyl cellulose; PVA, NaCMC, and HECEQ concentration: 5 mg/mL

* Statistically significant reduction of pH value compared to DPBS (*p* < 0.05)

#### Dynamic light scattering

DLS was employed to assess changes in the hydrodynamic diameter and dispersity of mucin assemblies in the presence of CPC (50 µg/mL), thymol (100 µg/mL), their combinations, and selected polymers. Mucin alone exhibited a mean hydrodynamic diameter of 426.7 ± 20.6 nm with a relatively high polydispersity index (PDI, 0.394 ± 0.006), reflecting the heterogeneous nature of the suspension.

**Fig. 5:**
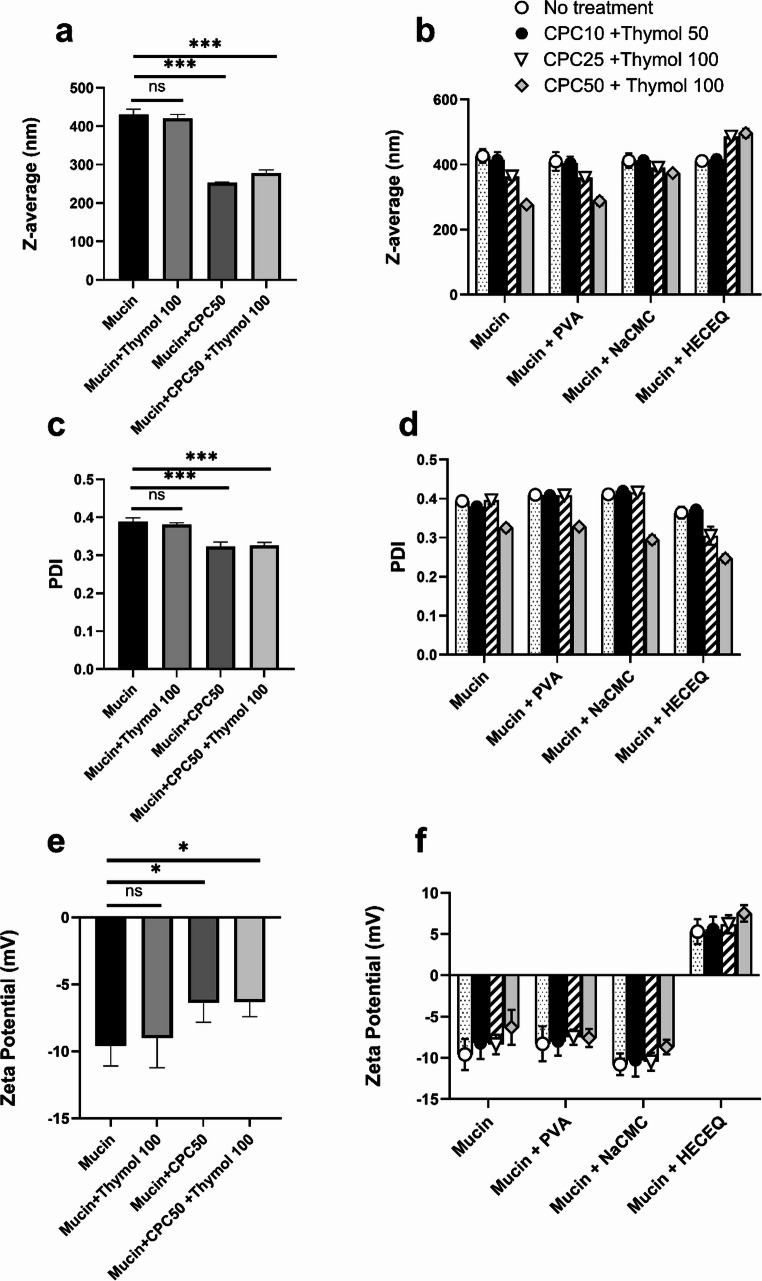
Interaction of antiseptic agents and polymers with mucin. (**a**) Changes in mucin particle size (Z-average) in DPBS alone or in the presence of 100 µg thymol/mL, 50 µg CPC/mL, or their combination. (**b**) Z-average of mucin particles in the presence of polymers, with or without the CPC–thymol combination. (**c**) Polydispersity index (PDI) of mucin in DPBS alone or with 100 µg thymol/mL, 50 µg CPC/mL, or their combination. (**d**) PDI of mucin in polymer-containing suspensions, with or without the CPC–thymol combinations. (**e**) Zeta potential of mucin in DPBS alone or in the presence of 100 µg thymol/mL, 50 µg CPC/mL, or their combination. (**f**) Zeta potential of mucin in polymer-containing suspensions, with or without the CPC–thymol combinations. Data represent mean ± SD (*n* = 3 independent experiments). Statistical analysis was performed using one-way ANOVA followed by Tukey’s test for pairwise comparisons among all groups (panels a, c, and e) and Dunnett’s test for comparisons against the control group (panels b, d, and f). Statistical significance is indicated in the graphs as *p < 0.05, **p < 0.01, and ***p < 0.001

As shown in Fig. 5a and c, the addition of 100 µg thymol/mL did not significantly alter either the hydrodynamic diameter or PDI of mucin (*p* > 0.05), indicating a lack of strong physicochemical interaction between thymol and mucin. In contrast, 50 µg CPC/mL markedly reduced both the mean hydrodynamic diameter (252.8 ± 13.6 nm) and PDI (0.363 ± 0.010) (*p* < 0.05 vs. mucin control), suggesting electrostatic complex formation between the cationic QAC and negatively charged mucin, resulting in a more homogeneous dispersion.

Regarding CPC-thymol combinations, a decrease in mucin particle size was observed depending on CPC concentration. At 50 µg CPC/mL and 100 µg thymol/mL, the reduction in size and PDI was comparable to that produced by 50 µg CPC/mL alone, whereas at 25 µg CPC/mL and 100 µg thymol/mL, the mean particle size decreased to 364 ± 11 nm (PDI 0.368 ± 0.011) (*p* < 0.05 vs. mucin control). At the lowest tested concentration (10 µg CPC/mL and 50 µg thymol/mL), no significant change in mucin particle size (416 ± 12 nm, PDI 0.391 ± 0.005) was observed relative to mucin alone (*p* > 0.05 vs. mucin control) (Fig. 5b and d).

Among the tested polymers, PVA (5 mg/mL) did not significantly affect mucin particle size (410 ± 29 nm) or dispersity (PDI 0.392 ± 0.013). However, in the presence of increasing CPC concentrations (10–50 µg/mL), mucin–PVA mixtures exhibited pronounced reductions in particle size (405–288 nm) and PDI (0.409–0.322). These reductions became statistically significant at CPC concentrations of 25 and 50 µg/mL (*p* < 0.05) (Fig. 5b and d), indicating polymer-mediated stabilization of smaller and more homogeneous complexes.

NaCMC (5 mg/mL) maintained mucin particle characteristics similar to the control, with no significant changes in particle size (412 ± 22 nm) or dispersity (PDI 0.397 ± 0.009) compared with mucin alone (*p* > 0.05). Addition of CPC–thymol induced only modest reductions in particle size (414–374 nm) compared with PVA-containing systems, suggesting reduced electrostatic interaction between CPC and mucin, potentially due to partial neutralization of CPC’s positive charge by NaCMC. Despite the relatively small magnitude of change, reductions in particle size became statistically significant at CPC concentrations of 50 µg/mL (*p* < 0.05). PDI values decreased progressively from 0.420 to 0.299 with increasing CPC concentrations (10–50 µg/mL), with a statistically significant reduction observed at 50 µg/mL (*p* < 0.05), indicating that the system became increasingly monodisperse as CPC concentration increased (Fig. 5b and d).

HECEQ (5 mg/mL) exhibited distinct behaviour: mucin combined with HECEQ alone displayed similar size and dispersity to native mucin (411 ± 13 nm, PDI 0.391 ± 0.01) (*p* > 0.05 vs. mucin control). However, in the presence of 25 and 50 µg CPC/mL and 100 µg thymol/mL, the mean particle size increased significantly compared to mucin control (486.2 ± 5.9 and 497 ± 7.0 nm, respectively) (*p* < 0.05), while PDI values decreased (0.305 ± 0.02 and 0.247 ± 0.01, respectively) (*p* < 0.05 vs. mucin control), suggesting the formation of larger, more uniform complexes (Fig. 5b and d).

#### Zeta potential

Zeta potential analysis confirmed that mucin particles carried a net negative surface charge (–9.6 ± 1.5 mV) in DPBS (Fig. 5e). Addition of 100 µg thymol/mL did not alter this charge (*p* > 0.05 vs. mucin control), whereas 50 µg CPC/mL significantly reduced the magnitude of the negative potential to − 6.38 ± 1.4 mV (*p* < 0.05 vs. mucin control), consistent with electrostatic binding and partial charge neutralization.

In CPC–thymol mixtures, the surface potential shifted toward neutrality in a CPC concentration–dependent manner, reaching − 8.2 ± 1.9 mV, − 8.4 ± 1.2 mV, and − 6.3 ± 2.1 mV at 10, 25, and 50 µg CPC/mL, respectively (Fig. 5f). In the presence of PVA or NaCMC, mucin retained its overall negative charge (–8.3 ± 2.1 mV and − 10.8 ± 1.3 mV, respectively), and the addition of CPC–thymol caused no significant deviation (–10.5 to − 7.5 mV).

Conversely, mucin combined with HECEQ exhibited a positive surface potential (+ 5.3 ± 1.5 mV), reflecting the cationic nature of the polymer. Co-incubation with CPC further increased this positive potential, reaching + 5.6 ± 1.5 mV, + 6.2 ± 1.1 mV and + 7.5 ± 1.0 mV at 10 µg/mL, 25 µg and 50 µg CPC per mL, respectively (*p* > 0.05 vs. mucin + HECEQ control).

## Discussion

### Virucidal activity and mechanisms of individual compounds

CPC and BZT, two widely used quaternary ammonium compounds (QACs), exhibited strong virucidal activity against both IAV and SARS-CoV-2, with higher potency against SARS-CoV-2. Previous studies have shown that CPC-containing mouthwashes at 0.05–0.1% can inactivate IAV by approximately 90–99.9% (~ 1–3 log₁₀) within 2 min [1] and achieve ≥ 4 log₁₀ reductions in SARS-CoV-2 titre within 30 s [11, 12]. In the present study, viral inactivation was achieved at lower CPC concentrations, which is likely influenced by the longer exposure time (30 min) used in our assays compared with previously reported short-contact conditions. Longer exposure times were included to explore sustained virucidal activity under controlled in vitro conditions relevant to potential mucoadhesive formulations, rather than to replicate immediate-contact antiseptic use. Data on BZT remain limited, and the present results therefore provide new evidence of its direct virucidal activity.

The antiviral mechanisms of QACs are considered to be concentration-dependent: at higher levels, CPC disrupts viral lipid envelopes through electrostatic and hydrophobic interactions [8, 13], while at lower levels, it can destabilize viral proteins such as SARS-CoV-2 spike (S), impairing receptor binding [10, 14].

Thymol, a phenolic monoterpene derived from thyme oil, also demonstrated direct virucidal activity against both viruses. Previous studies described both antiviral effects through intracellular targets and direct virucidal effects via disruption of viral particles [15, 16]. Mechanistically, thymol’s amphiphilic nature may enable insertion into viral lipid bilayers, increasing membrane fluidity and permeability, while hydrophobic interactions with capsid proteins can destabilize conformational integrity, rendering the virion non-infectious [16, 17]. The current findings confirm that thymol alone can directly inactivate enveloped respiratory viruses, albeit at higher concentrations compared to QACs.

#### Enhanced virucidal effects of QAC-thymol combinations

The most striking outcome was the strong enhancement of virucidal activity when QACs were combined with thymol. To the best of our knowledge, this is the first report demonstrating that thymol can potentiate the virucidal efficacy of QACs against enveloped respiratory viruses. Combinations of CPC or BZT with thymol achieved ≥ 4 log₁₀ reductions in viral titre at concentrations several-fold lower than those required for either agent alone, with rapid virucidal activity observed within 2 min for some combinations.

Although both CPC and thymol exert membrane-disruptive effects, their mechanisms are distinct and potentially complementary. QACs destabilize viral envelopes through electrostatic interactions with anionic phospholipids and denaturation of surface glycoproteins, whereas thymol inserts into lipid bilayers, perturbing hydrophobic packing and increasing membrane fluidity and permeability. Thymol-induced lipid disorder may facilitate deeper insertion of QACs, while QAC-mediated protein denaturation could expose additional hydrophobic domains for thymol interaction. Together, these cooperative disruptions at lipid–protein interfaces likely underlie the rapid and irreversible virion collapse observed.

Pedreira et al. (2024) also reported synergistic effects between QACs and essential oil constituents such as eugenol and carvacrol against *Escherichia coli* and *Bacillus cereus*, which they attributed to the formation of mixed QAC–phenolic dimers or micellar aggregates that enhance membrane penetration and intracellular access [18]. Although micelle formation is unlikely in the present study as the concentrations of QACs used were below the critical micelle concentration, the formation of transient dimers cannot be excluded and may help to enhance virucidal potency. Nevertheless, further studies are warranted to elucidate the molecular basis of this potentiated activity.

Importantly, the potentiated virucidal activity translated into reduced cytotoxicity: effective virucidal concentrations were substantially below the thresholds that impaired host cell viability, suggesting a potentially improved therapeutic window and favourable biocompatibility profile in this in vitro system.

### Differential susceptibility of SARS-CoV-2 and IAV

SARS-CoV-2 was consistently more susceptible to CPC, BZT, and thymol than IAV, requiring lower concentrations for inactivation. Structural and compositional differences between the two viruses may partly explain this pattern. SARS-CoV-2 acquires its envelope from the ER–Golgi intermediate compartment, which is enriched in phosphatidylserine and phosphatidylinositol and relatively poor in cholesterol and sphingolipids, resulting in a more fluid bilayer [12]. By contrast, IAV buds from the plasma membrane, a cholesterol-rich environment that produces a more rigid envelope [4]. Previous research has demonstrated that cholesterol-containing membranes remain largely impermeable to CPC compared with phospholipid-dominant membranes. The increased stiffness of cholesterol-rich bilayers restricts CPC insertion and limits its translocation across the membrane [19]. In addition, SARS-CoV-2 virions display ~ 20–40 spike trimers per particle, whereas IAV is densely coated with ~ 340 hemagglutinin and 24 neuraminidase molecules, which may hinder antiseptic access to envelope lipids [20, 21]. The relatively sparse surface proteins and more fluid lipid environment may increase SARS-CoV-2’s vulnerability to both lipid- and protein-targeting antiseptics, explaining its heightened sensitivity compared to IAV in this study.

Additionally, our findings demonstrate that the virucidal activity of CPC and BZT differed between the two viruses, with BZT showing stronger effects against IAV, while CPC was more potent against SARS-CoV-2. The molecular weights of the two QACs used were similar: CPC is 358 g/mol and BZT is 448.1 g/mol. This means that at 100 µg/mL, CPC is 279.4 µM whilst BZT is 223.2 µM and at 12.5 µg/mL CPC is 34.9 µM and BZT is 27.9 µM. The different activity trends displayed across the two viruses by each QAC suggest that differences in virucidal potency were not primarily due to molar disparities but rather reflect QAC-virus-specific interactions with envelope composition and protein architecture.

### Impact of interfering substances: mucin and polymers

We also evaluated the effect of an interfering substance, i.e. mucin, and some selected polymers on the virucidal activity of QACs, thymol and their combinations. Porcine gastric mucin (type II) was employed as a model system because it is affordable, readily available, and provides standardized material with reduced batch-to-batch variability compared to human salivary mucins [22]. Structurally, porcine gastric mucin shares several glycan motifs with human salivary mucins, including sialic acid, fucose, and N-acetyl galactosamine, making it a widely used surrogate in drug–mucin interaction studies [23]. To prepare mucin for assays, solutions were autoclaved at 121 °C for 15 min; previous work has shown that autoclaving does not significantly disrupt mucin’s structural integrity [24]. Centrifugation removed only ~ 7% of the intial mucin mass, confirming that the supernatant fraction used in our assays still contained large amounts of mucin that were in the known physiological range of human saliva (1–3 mg/mL) [25] and is consistent with artificial-saliva formulations previously used to replicate the biochemical and rheological properties of natural saliva [26].

**Fig. 6:**
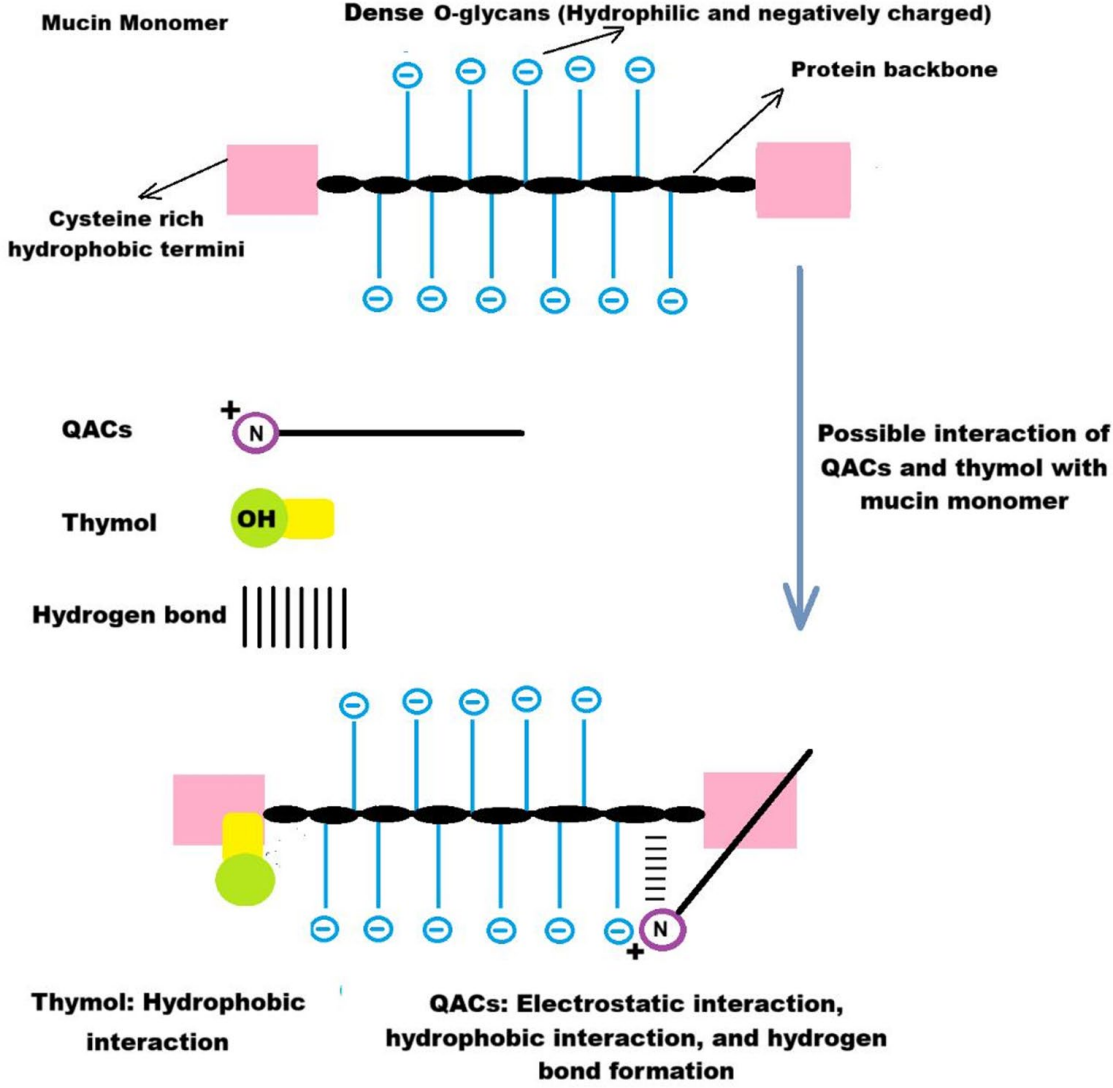
Schematic illustration showing the proposed interactions between mucin, quaternary ammonium compounds (QACs), and thymol, highlighting possible electrostatic, hydrophobic, and hydrogen-bonding interactions between QACs and mucin, and hydrophobic interactions between thymol and mucin

The pronounced attenuation of CPC and BZT activity by mucin, contrasted with the relative preservation of thymol efficacy, may be explained by multivalent interactions between QACs and mucin that combine electrostatic initiation with hydrophobic and hydrogen-bonding stabilization (Fig. 6). Mucins are large amphiphilic glycoproteins: their central domains are extensively O-glycosylated, enriched with sialic acid and sulphate groups, conferring a strong negative charge and hydrophilicity, whereas the terminal cysteine-rich domains are sparsely glycosylated and expose hydrophobic residues [24].

CPC and BZT are amphiphilic surfactants with a positively charged quaternary ammonium headgroup and a long hydrophobic alkyl tail [19]. This complementary architecture enables multiple binding modes with mucin. Electrostatic attraction likely initiates binding between the cationic headgroups and negatively charged mucin glycans, while hydrophobic insertion of the alkyl tails into mucin’s hydrophobic domains, together with hydrogen bonding to peptide backbones, stabilizes the interaction. Importantly, NaCMC, a purely anionic and highly hydrated polysaccharide lacking hydrophobic domains [27], did not reproduce mucin’s inhibitory effect on CPC, indicating that charge complementarity alone does not lead to effective surfactant sequestration. This observation suggests that hydrophobic and hydrogen-bonding contributions are critical determinants of mucin–QAC interactions and underlie the pronounced attenuation of CPC and BZT virucidal activity in the presence of mucin.

By contrast, thymol activity was largely preserved in the presence of mucin. This difference likely reflects thymol’s distinct mode of action: as a small, neutral hydrophobic phenolic, thymol disrupts viral envelopes mainly through direct insertion into lipid bilayers rather than through charge-mediated binding [17]. Because thymol does not carry a positive charge, it is not strongly attracted to mucin’s negatively charged glycans, and any hydrophobic interactions with mucin domains are relatively weak [28]. Consequently, mucin is less able to sequester thymol, leaving its effective free concentration intact and its virucidal activity largely unaffected.

DLS and zeta potential measurements reinforced this model. Mucin exhibited negatively charged, heterogeneous assemblies in buffer. Addition of 50 µg CPC/mL significantly reduced particle size, polydispersity, and zeta potential, indicating strong interaction between CPC and mucin, consistent with partial charge neutralization and compaction of mucin assemblies [29]. This strong association may explain the pronounced attenuation of CPC’s virucidal activity in the presence of mucin. In contrast, addition of 100 µg thymol/mL did not cause measurable shifts in mucin’s particle size, PDI, or zeta potential, consistent with the partially preserved activity of thymol. For the CPC–thymol combinations, at 25 µg CPC/mL, mucin effectively depleted virucidal activity (~ 1 Δlog_10_ TCID_50_/mL), whereas at 50 µg CPC/mL (~ 4 Δlog_10_ TCID_50_/mL), antiviral efficacy was largely maintained despite mucin. Two mechanisms may explain this concentration dependence: (1) higher CPC levels may saturate mucin binding sites, leaving a greater fraction of free CPC available for virucidal action; and (2) CPC-induced compaction may reorganize mucin assemblies such that hydrophobic pockets become less accessible and bound CPC is more readily exchangeable. Both explanations are consistent with the observed reduction in mucin particle size, decreased PDI, and partial preservation of virucidal activity at higher CPC concentrations.

The presence of polymers partially preserved the virucidal activity of the CPC–thymol combination in mucin-containing systems, although the extent of preservation varied among polymers. As shown in Fig. 3b–d, supplementation with PVA, NaCMC, or HECEQ consistently resulted in higher log₁₀ reductions in viral titre compared with mucin alone for both IAV and SARS-CoV-2, indicating that these polymers can, at least partially, mitigate the inhibitory effects of mucin on antiseptic activity. Among the polymers tested, NaCMC produced the greatest enhancement of virucidal efficacy across all viruses–concentration combinations.

PVA, NaCMC, and HECEQ are hydrophilic, linear polymers bearing neutral, negative, and positive surface charges, respectively. Based on visual observation, the relative viscosity of 0.5 mg polymer solutions per mL ranked as HECEQ > NaCMC > PVA. No clear correlation was observed between medium viscosity and the rank order of protective effects (NaCMC > PVA ≥ HECEQ), suggesting that polymer-mediated preservation of virucidal activity is unlikely to be viscosity-dependent. Instead, the protective effect may arise from reduced sequestration of CPC–thymol through steric hindrance, altered binding kinetics, or weak competitive interactions with mucin, thereby maintaining a fraction of free CPC available for viral inactivation.

In the case of NaCMC, its anionic carboxymethyl groups may preferentially interact with cationic quaternary ammonium compounds [27], forming transient complexes that limit CPC binding to mucin while still permitting interaction with viral envelopes. This mechanism could explain the consistently higher viral titre reductions observed with NaCMC compared with PVA and HECEQ. Supporting this hypothesis, DLS measurements revealed a reduced extent of CPC-induced mucin particle size compaction in the presence of NaCMC, indicating attenuated CPC–mucin interactions, which is consistent with the observed superior preservation of virucidal activity.

Overall, these findings suggest that while all tested polymers contribute to preserving virucidal activity in mucin-containing systems, polymer chemical functionality plays a critical role in determining the magnitude of this effect. The superior performance of NaCMC highlights its potential as a formulation excipient for mucoadhesive oral delivery systems designed to retain antiviral efficacy under these in vitro mucin-containing conditions.

Although HECEQ may reduce CPC–mucin interactions through competitive binding to negatively charged mucin domains, the distinct DLS and zeta potential profiles observed for HECEQ provide a possible explanation for its comparatively weaker preservation of virucidal activity. Charge inversion of mucin and the formation of larger, more uniform complexes upon addition of HECEQ and CPC suggest strong associative interactions that may promote sequestration of CPC within polymer–mucin assemblies, thereby limiting its availability for viral inactivation.

## Limitation of the study

This study has several limitations that should be acknowledged. First, following virucidal exposure, samples were subjected to serial ten-fold dilution in ice-cold medium prior to titration. Based on cytotoxicity controls, the 10⁻² dilution was used as the lowest valid dilution for CPE assessment, corresponding to residual compound concentrations substantially below those associated with measurable antiviral activity. Although a formal neutralization assay was not performed, these findings, together with cytotoxicity controls, suggest that residual compound activity during titration is unlikely to significantly affect viral titre measurements.

Second, the cytocompatibility assays were conducted using MDCK-II and VAT cell lines, which are non-human models. While these systems provided valuable insights into the comparative biocompatibility of individual compounds and their combinations, further investigations in more physiologically relevant oral epithelial or organotypic models are required to confirm safety and translational applicability.

Third, porcine gastric mucin was employed as a surrogate for human salivary mucin in evaluating mucin–antiseptic interactions. Structurally, both are large, heavily glycosylated proteins that share key glycan motifs, including sialic acid, fucose, and N-acetyl galactosamine, making porcine mucin a reasonable model for preliminary in vitro experiments. However, important differences remain. Human saliva predominantly contains MUC5B and MUC7, whereas porcine gastric mucin is enriched in MUC5AC, and glycosylation profiles vary across species and tissue sources. Thus, while porcine mucin provides an approximation of mucosal interactions, confirmatory studies employing human salivary mucins or ex vivo saliva would strengthen the physiological relevance of these findings.

In addition, DLS and zeta potential measurements were performed after dilution and at 25 °C, which may influence interaction equilibria; therefore, these data are interpreted comparatively rather than as exact in situ physicochemical states.

## Conclusion

This study shows that CPC, BZT, and thymol exhibit potent virucidal activity against two enveloped respiratory viruses, IAV and SARS-CoV-2. Notably, we show that thymol enhances the virucidal efficacy of QACs, enabling ≥ 4 log₁₀ reductions in viral titre under the tested in vitro conditions at substantially lower QAC concentrations than required for individual compounds. This enhanced activity accelerates viral inactivation, achieving reductions of approximately 4 log₁₀ within minutes, while also improving cytocompatibility.

The presence of mucin, a major salivary glycoprotein, attenuated QAC activity, whereas thymol largely retained its efficacy. Selected polymers partially preserved virucidal activity under mucin-rich conditions, although to varying extents, highlighting the importance of evaluating antiviral agents in more complex and physiologically representative systems. These findings underscore how mucosal components and macromolecules can modulate antiviral efficacy. These findings may inform future strategies to maintain antiviral activity in the presence of mucin; however, further studies in more physiologically relevant models are required.

## Supplementary Information

Below is the link to the electronic supplementary material.

Supplementary Material 1 (DOCX 24.7 K)

## Data Availability

The data generated and analysed in the present study are included in this manuscript. Additional information is available from the corresponding author upon reasonable request.
