# Supplementary material for: Enhanced virucidal activity of quaternary ammonium compound–thymol combinations: influence of mucin and mucoadhesive polymers
Source: Arch Virol. 2026 Jun 18;171(7):215. doi: 10.1007/s00705-026-06672-8 (PMC13275772; doi:10.1007/s00705-026-06672-8)
Supplement: Supplementary file 1 — Supplementary Material 1 (DOCX 24.7 K) [file 705_2026_6672_MOESM1_ESM.docx]

Table S1. Virucidal activity of CPC–thymol combinations in DPBS with and without polymers

| Virus | Combination | Polymer condition | CPC (µg/mL) | Thymol (µg/mL) | Δlog₁₀ TCID₅₀/mL |
| --- | --- | --- | --- | --- | --- |
| IAV | CPC–thymol | DPBS | 25 | 100 | ≥4.4 |
| IAV | CPC–thymol | DPBS + PVA | 25 | 100 | ≥4.4 |
| IAV | CPC–thymol | DPBS + NaCMC | 25 | 100 | ≥4.4 |
| IAV | CPC–thymol | DPBS + HECEQ | 25 | 100 | ≥4.4 |
| IAV | CPC–thymol | DPBS | 50 | 100 | ≥4.4 |
| IAV | CPC–thymol | DPBS + PVA | 50 | 100 | ≥4.4 |
| IAV | CPC–thymol | DPBS + NaCMC | 50 | 100 | ≥4.4 |
| IAV | CPC–thymol | DPBS + HECEQ | 50 | 100 | ≥4.4 |
| SARS-CoV-2 | CPC–thymol | DPBS | 10 | 50 | ≥4.0 |
| SARS-CoV-2 | CPC–thymol | DPBS + PVA | 10 | 50 | ≥4.0 |
| SARS-CoV-2 | CPC–thymol | DPBS + NaCMC | 10 | 50 | ≥4.0 |
| SARS-CoV-2 | CPC–thymol | DPBS + HECEQ | 10 | 50 | ≥4.0 |
